# Prevalencia y caracterización de la coinfección por el virus de la inmunodeficiencia humana en pacientes hospitalizados con tuberculosis en un hospital de referencia de Bogotá

**DOI:** 10.7705/biomedica.7664

**Published:** 2025-08-11

**Authors:** Gean Carlo Puentes-Ariza, Sugeich Meléndez-Rhenals, Laura Cristina Nocua-Báez, Hugo Páez-Ardila

**Affiliations:** 1 Departamento de Medicina Interna, Facultad de Medicina, Universidad Nacional de Colombia, Bogotá, D.C., Colombia Universidad Nacional de Colombia Universidad Nacional de Colombia Bogotá D.C. Colombia; 2 Servicio de Medicina Interna, Hospital Universitario Nacional de Colombia, Corporación Salud UN, Bogotá, D.C., Colombia Hospital Universitario Nacional de Colombia Bogotá D.C Colombia; 3 Servicio de Infectología, Hospital Simón Bolívar, Bogotá, D.C., Colombia Hospital Simón Bolívar Bogotá D.C Colombia

**Keywords:** tuberculosis pulmonar, HIV, infecciones oportunistas, síndrome de inmunodeficiencia adquirida, Tuberculosis, pulmonary, HIV, opportunistic infections, acquired immunodeficiency syndrome

## Abstract

**Introducción.:**

La tuberculosis es la principal causa de muerte en los pacientes con infección por el virus de la inmunodeficiencia humana. La coinfección de *Mycobacterium tuberculosis* y HIV es muy frecuente.

**Objetivo.:**

Establecer la prevalencia de la infección por HIV en pacientes hospitalizados con tuberculosis, y determinar sus características y desenlaces.

**Materiales y métodos.:**

Se realizó un estudio retrospectivo, de corte transversal, en pacientes con tuberculosis -pulmonar o extrapulmonar- y coinfección por HIV, hospitalizados en una institución de referencia de Bogotá entre el 2019 y el 2021.

**Resultados.:**

En el grupo de los 102 pacientes con tuberculosis, la prevalencia de infección por HIV fue del 52,3% (54). Entre estos 54, 24 pacientes (44,4%) tuvieron confirmación microbiológica o histopatológica de la tuberculosis y, 19 (35,2 %), infección por VIH *de novo*. En los 54 coinfectados, la mediana de la edad fue de 38 años (RIC: 29-42). El 79,6 % (43/54) fueron hombres. La mediana del número de linfocitos T CD4^+^ fue de 59 células/ μl (RIC: 32-120), y el 72,2 % (39/54) tenía menos de 200 células/μl. El 31,4 % (17/54) de los pacientes con antecedente de infección por HIV recibía terapia antirretroviral. En cuanto a la forma clínica, la tuberculosis fue pulmonar en el 51,9 % (28/54) y extrapulmonar en el 48,1 % (26/54) de los pacientes. La tuberculosis extrapulmonar fue meníngea (29,6 %), miliar (12,9 %), pleural (3,7 %) y peritoneal (3,7 %). Hubo 33,3 % de mortalidad intrahospitalaria, asociada con el número de linfocitos T CD4^+^ (p < 0,05), el diagnóstico *de novo* de HIV (p < 0,04) y la presencia de tuberculosis meníngea (p < 0,03).

**Conclusión.:**

La coinfección de *Mycobacterium tuberculosis* y HIV es frecuente y se relaciona con una inmunosupresión avanzada, por lo que debe hacerse una búsqueda activa de la infección con HIV en estos casos. La tuberculosis meníngea fue la forma extrapulmonar más frecuente y se asoció con mortalidad.

## Abstract (en)

**Introduction.:**

Tuberculosis is the leading cause of death in patients with human immunodeficiency virus infection; tuberculosis-HIV coinfection is highly prevalent.

**Objective.:**

To establish the prevalence of HIV infection in patients hospitalized with tuberculosis and determine their characteristics and outcomes.

**Materials and methods.:**

We conducted a retrospective cross-sectional study in patients diagnosed with pulmonary or extrapulmonary tuberculosis and HIV coinfection, hospitalized in a referral institution in Bogotá between 2019 and 2021.

**Results.:**

The prevalence of HIV infection in 102 patients with tuberculosis was 52.3% (54/102). Among them, 24 patients (44.4%) had microbiological or histopathological confirmation of tuberculosis infection; and 19 (35.2%) were recently diagnosed with an HIV infection. The median age of the 54 coinfected patients was 38 years (IQR: 29-42); 79.6% (43/54) were men. The median CD4+ T-cell count was 59 cells/μL (IQR: 32-120), and 72.2% (39/54) had a CD4+ count of less than 200 cells/μL; 31.4% (17/54) of patients with a history of HIV infection were receiving antiretroviral therapy. Regarding tuberculosis clinical presentation, 51.9% (28/54) had pulmonary tuberculosis, and 48.1% (26/54) had extrapulmonary tuberculosis. The extrapulmonary tuberculosis forms were meningeal (29.6%), miliary (12.9%), pleural (3.7%), and peritoneal (3.7%). The in-hospital mortality was 33.3%, associated with a low CD4^+^ count (p < 0.05), recent HIV diagnosis (p < 0.04), and the presence of meningeal tuberculosis (p < 0.03).

**Conclusion.:**

The frequency of tuberculosis-HIV coinfection is high and related to an advanced degree of immunosuppression. Therefore, we recommend an active search for coinfection. Meningeal tuberculosis was the most frequent extrapulmonary form and was associated with mortality.

La infección por el virus de la inmunodeficiencia humana (*Human Immunodeficiency Virus*, HIV) es una de las principales causas de morbilidad y mortalidad en el mundo ^1^^,^^2^. En esta enfermedad, hay disminución del número y la función de los linfocitos T CD4^+^, lo que afecta al sistema inmunológico celular en general y, por lo tanto, predispone a varias complicaciones, tanto infecciosas como oncológicas ^2^^,^^3^. La infección por *Mycobacterium tuberculosis* es la primera causa de muerte por un solo agente infeccioso a nivel mundial, con 1,25 millones de personas fallecidas en 2023 ^4^.

La tuberculosis es la infección oportunista más frecuente y la principal causa de muerte en personas con HIV ^5^. La infección por HIV es el factor de riesgo individual más importante para el desarrollo de la tuberculosis, ya que incrementa de 20 a 30 veces la probabilidad de presentar la infección activa. Además, se asocia con las formas clínicas más graves de la enfermedad ^6^^-^^8^, incluyendo los casos de tuberculosis farmacorresistente ^9^. El grado de inmunosupresión influye en este riesgo: por ejemplo, las personas con valores de linfocitos T CD4^+^ inferiores a 100 células/ml, tienen una probabilidad diez veces mayor de presentar la enfermedad que aquellos con más de 500 células/ml ^10^.

La coinfección de *M. tuberculosis* y HIV tiene una presentación clínica variable; es más probable que se presenten formas extrapulmonares de la enfermedad, si hay un mayor grado de inmunosupresión. La linfadenitis tuberculosa es la forma más frecuente ^6^.

En Colombia, los datos reportados sobre las características de esta coinfección no son recientes. Según los registros del Sistema Nacional de Vigilancia en Salud Pública (SIVIGILA) del 2021, de los 14.060 casos de todas las formas de tuberculosis, el 12,1 % tenía coinfección por HIV ^11^.

La Organización Panamericana de la Salud (OPS), en su reporte sobre coinfección por *M. tuberculosis* y HIV del 2020 en las Américas, documentó un estimado de 291.000 casos nuevos de tuberculosis y una frecuencia de coinfección por HIV del 10 %. Del total de muertes por tuberculosis en el 2020, el 29 % correspondió a personas con dicha coinfección. Además, el resultado de la serología para HIV se conocía en 79 % de los casos nuevos de tuberculosis en los países incluidos en el informe ^12^. En el 2023, de los 10,8 millones de personas con tuberculosis incidente a nivel mundial, el 6,1 % correspondió a pacientes con infección por HIV. Ese mismo año, entre los 1,25 millones de muertes por tuberculosis, 161.000 pacientes presentaban la coinfección ^4^.

El objetivo de este estudio fue determinar la prevalencia de la infección por HIV en pacientes con tuberculosis pulmonar o extrapulmonar hospitalizados en una institución de referencia de Bogotá (Colombia), caracterizar la población con la coinfección y establecer los factores asociados con la mortalidad en estos casos.

## Materiales y métodos

### Diseño

Este es un estudio de diseño retrospectivo, de corte transversal, en el que se estimó la prevalencia de infección por HIV en los pacientes con diagnóstico de tuberculosis pulmonar o extrapulmonar. Se determinaron las características sociodemográficas y clínicas, y se identificaron los resultados de interés en aquellos con coinfección por HIV y *M. tuberculosis*. Los pacientes estaban internados en el Hospital Simón Bolívar de Bogotá, una institución pública de referencia en la atención de pacientes con HIV. El periodo de estudio fue del 1° de enero del 2019 al 31 de diciembre del 2021.

### Población

Los criterios de inclusión fueron: pacientes adultos (mayores de 18 años), diagnosticados durante la hospitalización con tuberculosis activa (pulmonar o extrapulmonar), según las directrices del Programa Nacional de Tuberculosis ^13^, que hubieran requerido iniciar tratamiento antituberculoso y que tuvieran confirmación de coinfección con HIV, conocida o de *novo*. El criterio de exclusión fue el diagnóstico de tuberculosis latente.

### Procedimiento

Después de la aprobación por el comité de ética institucional, se hizo una búsqueda inicial en el registro de pacientes con diagnóstico de sospecha o confirmación de infección por tuberculosis incluidos en el programa institucional de tuberculosis. Se excluyeron los pacientes a quienes se les descartó la infección. A continuación, aquellos con infección confirmada por HIV (previa o de novo) se seleccionaron para continuar con la revisión detallada de la historia clínica electrónica en el sistema *Servinte Clinical Suite* del hospital.

Los datos obtenidos se consignaron en el formulario de recolección de la información del estudio. El 15 % de las historias clínicas se seleccionó al azar para la verificación y revisión de los datos extraídos por parte de un investigador.

### Análisis estadístico

Se utilizó el *software* Stata™, versión 15.0 ^14^, para el análisis estadístico. Se hizo un análisis descriptivo univariado. Se usaron frecuencias relativas y absolutas para las variables categóricas. La normalidad de las variables continuas se evaluó mediante el valor del sesgo de la variable, los diagramas de cajas y bigotes e histogramas. Las variables continuas no se ajustaron a una distribución normal, por lo que se describieron con la mediana y el rango intercuartílico (RIC).

Se utilizó un análisis bivariado entre la variable de exposición (HIV *de novo*) y la variable de resultado (tuberculosis pulmonar o extrapulmonar), así como entre la variable de exposición, las covariables y el desenlace, para determinar la asociación o la independencia entre ellas. Como las variables no contaban con una distribución normal, se usó la prueba de χ^2^ para las variables categóricas y la prueba de suma de rangos de Wilcoxon (U de Mann-Whitney) para las variables continuas.

Se llevó a cabo un análisis bivariado exploratorio entre la variable de mortalidad intrahospitalaria y las demás covariables para determinar la asociación o la independencia entre ellas. Se hizo un subanálisis de los pacientes con confirmación microbiológica de infección por tuberculosis.

### Consideraciones éticas

Esta investigación se realizó siguiendo las consideraciones de la regulación colombiana (Resolución 8430 de 1993, artículo 11) ^15^. El estudio fue aprobado por el Comité de Ética en Investigación de la Subred Integrada de Servicios de Salud Norte E.S.E. El código del proyecto fue SNCEI-150 y la aprobación se encuentra en el Acta 54-CEI.

## Resultados

Se encontraron 102 casos de tuberculosis pulmonar o extrapulmonar. La coinfección por *M. tuberculosis* y HIV se registró en 54 casos, lo que correspondió a una prevalencia del 52,3 %. Entre ellos, 24 casos (44,4 %) tuvieron confirmación microbiológica o histopatológica de la infección por tuberculosis. Las características sociodemográficas de los pacientes incluidos se muestran en el cuadro 1. La mediana de edad fue de 38 años (RIC: 29-42 años); la mayoría estaba entre los 30 y los 39 años (33,3 %).

**Cuadro 1:** Características sociodemográficas de pacientes con coinfección por *Mycobacterium tuberculosis* y *Human Immunodeficiency Virus* (N = 54)

Variable	Categoría	Total
Edad en años (RIC)		38 (29-42)
Sexo [n (%)]	Femenino	10 (18,52)
	Masculino	43 (79,63)
	Transgénero	1 (1,85)
Nacionalidad [n (%)]	Colombiana	39 (72,22)
	Venezolana	15 (27,78)
Escolaridad [n (%)]	Sin escolaridad	2 (3,7)
	Primaria	17 (31,48)
	Bachillerato	20 (37,04)
	Técnico	1 (1,85)
	Pregrado	3 (5,56)
	Sin dato	11 (20,37)
Orientación sexual [n (%)]	Heterosexual	29 (53,7)
	Hombres que tienen sexo con hombres	11 (20,37)
	Transexual	1 (1,85)
	Sin dato	13 (24,07)

RIC: rango intercuartílico

**Figura 1:**
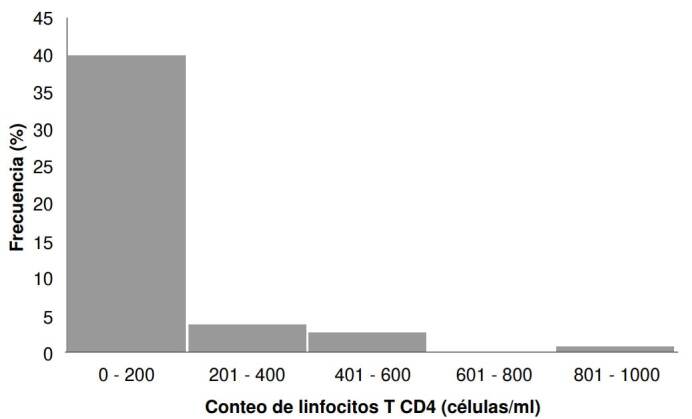
Frecuencias relativas de la variable del número de linfocitos T CD4+ en la población de estudio

La carga viral de HIV en la población estudiada tuvo una mediana de 185.349 copias de ARN/ml (RIC: 42.656-462.000). Sin embargo, este dato no estuvo disponible en el 30 % de los casos, aproximadamente. Además, se observaron medidas de dispersión amplias y una distribución que no cumplía con los supuestos de normalidad. En el 87 % de los pacientes incluidos, se registró el número de linfocitos T CD4^+^; se encontró una mediana de 59 células/ciru (RIC: 32-120), el valor máximo fue de 982 células/μl y el valor mínimo fue de 2 células/μl (figura 1). Las características clínicas de la población del estudio se muestran en el cuadro 2.

**Cuadro 2:** Características clínicas de los pacientes con coinfección por *Mycobacterium tuberculosis y Human Immunodeficiency Virus* (N = 54)

Variable	Categoría	Total
Carga viral (n = 39), copias de ARN/ml (RIC)		185.349 (42.656-462.000)
Número de linfocitos T CD4+ (n =	47), células/μl (RIC)	59 (32-120)
Categoría según número de	Estado 1 (> 500 células/μl)	3 (5,56)
CD4+ [n (%)]	Estado 2 (200-499 células/μl)	2 (9,26)
	Estado 3 (< 200 células/μl)	39 (72,22)
	Sin dato	7 (12,96)
Diagnóstico de HIV [n (%)]	Reciente	19 (35,19)
	Previo	35 (64,81)
Otra infección oportunista [n (%)]	Sí	30 (55,56)
	No	24 (44,44)
Tipo de tuberculosis [n (%)]	Pulmonar	28 (51,85)
	Extrapulmonar	26 (48,15)
Localización [n (%)]	Pulmonar	23 (42,59)
	Meníngea	16 (29,63)
	Miliar	4 (7,41)
	Miliar y pulmonar	3 (5,56)
	Peritoneal	2 (3,7)
	Pleural	2 (3,7)
	Pericárdica	1 (1,85)
	Intestinal	1 (1,85)
	Meníngea y pulmonar	1 (1,85)
	Ganglionar y pulmonar	1 (1,85)
Tratamiento antituberculoso	Sí	12 (22,22)
previo [n (%)]	No	42 (77,78)
Muerte intrahospitalaria [n (%)]	Sí	18 (33,3)
	No	36 (66,7)

Según el número de linfocitos T CD4^+^ de las categorías de los *Centers for Disease Control and Prevention* (CDC) ^16^, 39 pacientes (72,2 %) fueron clasificados en estado 3 (< 200 células/μl). De los 54 pacientes con coinfección por *M. tuberculosis* y HIV, en 19 (35,2 %) se hizo *de novo* el diagnóstico de infección por HIV a partir de la sospecha de tuberculosis, mientras que en 35 (64,8 %) ya se conocía el antecedente de HIV. En el momento del hallazgo de la coinfección por *M. tuberculosis* y HIV, 30 pacientes (55,6 %) tuvieron otra infección oportunista; las más frecuentes fueron candidiasis (18,5 %), enfermedad por citomegalovirus (18,5 %) y sífilis (9,2 %). Doce pacientes (22,2 %) presentaron dos o más enfermedades oportunistas concomitantes.

Respecto a la localización de la tuberculosis, 26 pacientes (48,1 %) presentaron la forma extrapulmonar y 28 (51,9 %) presentaron la forma pulmonar. De estos últimos, cinco tenían compromiso extrapulmonar concomitante. Las formas clínicas de los casos de tuberculosis y su localización anatómica se muestran en el cuadro 2. Los hallazgos radiológicos más frecuentes en los 28 pacientes con tuberculosis pulmonar fueron: infiltrados nodulares o micronodulares (57,1 %), cavitaciones (32,1 %) u opacidades parenquimatosas en “árbol en gemación” (17,9 %).

La confirmación microbiológica de la infección por tuberculosis se hizo en 19 pacientes (35,2 %), se realizaron estudios histopatológicos en 5 casos (9,3 %) y se obtuvo reporte positivo de adenosina deaminasa (ADA) en líquidos corporales de 7 individuos (12,9 %). En 25 pacientes (46,3 %), se tuvieron en cuenta criterios clínicos y radiológicos para iniciar el tratamiento antituberculoso. El 22,2 % del total de pacientes incluidos (N = 54) había recibido tratamiento previamente.

En el grupo de pacientes con tuberculosis pulmonar, en 11 (39,3 %), el cultivo mostró crecimiento de *M. tuberculosis*; en 6 (21,4 %), la baciloscopia demostró bacilos ácido-alcohol resistentes; en 5 (17,9 %), la reacción en cadena de la polimerasa (PCR) mostró amplificación de ADN de *M. tuberculosis*; y en 4 (14,3 %), los hallazgos histopatológicos correspondieron a la enfermedad. En 10 casos (18,5 %), se indicó tratamiento antituberculoso con base en criterios clínicos y radiológicos.

En el análisis del subgrupo de 24 pacientes (44,4 %) con confirmación microbiológica o histopatológica de la infección por tuberculosis, 20 (83,3 %) fueron confirmados por baciloscopia, PCR o cultivo positivo, mientras 4 (16,7 %) presentaron hallazgos histopatológicos indicativos de la enfermedad. De estos pacientes,18 (75 %) desarrollaron tuberculosis pulmonar, entre los cuales 5 registraron formas extrapulmonares concomitantes. Los 6 restantes (25 %) presentaron compromiso exclusivamente extrapulmonar. Se recolectaron los datos de la sensibilidad a antibióticos de las cepas de *M. tuberculosis* aisladas en 12 casos (50 %): en 9 (75 %), no presentaron resistencia, en 2 (16,7 %), la tuberculosis se clasificó como monorresistente y, en 1 (8,3 %), como tuberculosis multifarmacorresistente. Siete (29,2 %) pacientes habían recibido tratamiento antituberculoso previamente. El número de linfocitos T CD4^+^ en este subgrupo, tuvo una mediana de 65,5 células/μl (RIC: 52,25-175,5) y la carga viral de HIV tuvo una mediana de 190.674 copias de ARN/ml (RIC: 63.583-386.296). La infección por HIV se diagnosticó *de novo* en 9 pacientes (37,5 %) y se registraron 3 muertes (12,5 %).

En el análisis por subgrupos según el tipo de diagnóstico de HIV, este fue *de novo* en 19 pacientes. En ellos, la mediana del número de linfocitos CD4+ fue de 53 células/μl (RIC: 37,5-62) y la mediana de carga viral fue de 324.928 copias de ARN/ml (RIC: 219.592-699.500). Once pacientes (57,9 %) desarrollaron tuberculosis extrapulmonar, 15 (78,9 %) presentaron otra infección oportunista y 10 (52,6 %) murieron durante la hospitalización.

**Figura 2:**
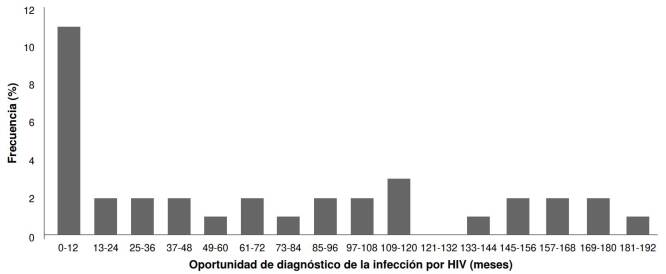
Frecuencias relativas del tiempo de oportunidad de diagnóstico de HIV durante el curso de la infección con tuberculosis

En los 35 pacientes con antecedente conocido de infección por HIV, la mediana del número de linfocitos T CD4+ fue de 66 células/μl (RIC: 45,51790) y la mediana de la carga viral fue de 70.145 copias de ARN/ml (RIC: 36.972-261.581,75); en tres pacientes, la carga fue indetectable. La mediana del tiempo de diagnóstico de la infección por HIV fue de 72 meses (RIC: 12120), con un valor mínimo de un mes y un valor máximo de 192 meses. En la mayoría de los casos, el diagnóstico se hizo el año anterior al hallazgo de la coinfección por tuberculosis (figura 2). En este subgrupo, 11 (31,4 %) pacientes se encontraban en tratamiento antirretroviral al momento del diagnóstico; en 15 de 35 (42,9 %), se identificó al menos otra enfermedad oportunista y, en 15 de 35 (42,9 %), se detectó tuberculosis extrapulmonar. Se registró mortalidad intrahospitalaria en 8 pacientes (22,9 %).

En total, se reportaron 18 muertes intrahospitalarias; en estos casos, la mediana de la edad fue de 39,5 años (RIC: 30,25-47), la del número de linfocitos T CD4+ fue de 53 células/μl (RIC: 40,5-60,25) y la de la carga viral de 324.928 copias de ARN/ml (RIC: 28.329-608.006). Trece (72,2 %) pacientes presentaron otra infección oportunista; 7 (38,9 %) tenían tuberculosis pulmonar; y 11 (61,1 %) desarrollaron tuberculosis extrapulmonar. La forma clínica más frecuente fue la meníngea (50 %).

Se hizo un análisis bivariado exploratorio, en el cual se tomó como variable de exposición a la infección por HIV *de novo* y como desenlace, la presencia de tuberculosis pulmonar. El resultado del análisis no encontró asociación estadísticamente significativa entre estas dos variables. En las comparaciones entre cada tipo de tuberculosis y las demás covariables, tampoco se encontró una asociación estadísticamente significativa. Dichas covariables incluyeron: sexo, edad, nacionalidad, escolaridad, orientación sexual, estado de inmunosupresión (según el número de linfocitos T CD4^+^), carga viral, tiempo de diagnóstico de la infección por HIV, tratamiento antirretroviral, diagnóstico de otras infecciones oportunistas, antecedente de tratamiento antituberculoso y mortalidad intrahospitalaria.

En el subanálisis de los pacientes con diagnóstico *de novo* por HIV, respecto a aquellos con diagnóstico previo, se encontró que las medianas de carga viral fueron significativamente diferentes (p < 0,015); las demás covariables estudiadas fueron independientes entre sí. En el análisis bivariado de los pacientes con diagnóstico *de novo* de infección por HIV con respecto a las demás covariables, hubo una asociación estadísticamente significativa con el resultado de mortalidad intrahospitalaria, tanto en casos pulmonares como extrapulmonares (p < 0,04). Esta asociación no se encontró en el grupo de los pacientes con infección previa por HIV.

Se hizo un análisis exploratorio entre los pacientes hospitalizados fallecidos y los sobrevivientes, y se encontró una asociación significativa con el número de linfocitos T CD4+ (p < 0,04) -mediante la prueba de Wilcoxon para variables continuas- y con la presencia de tuberculosis meníngea (p < 0,03) -mediante la prueba de χ^2^. No se encontró ninguna asociación con las variables de sexo, edad, nacionalidad, carga viral, terapia antirretroviral o infecciones oportunistas concomitantes.

## Discusión

En el presente estudio, se encontró una prevalencia de infección por HIV del 52,3 % (54/102) en pacientes con tuberculosis -confirmada por pruebas microbiológicas e histopatológicas o hallazgos clínicos o radiológicos- hospitalizados en una institución de referencia para personas con HIV en Bogotá, entre el 2019 y el 2021. La prevalencia de la infección por HIV en el grupo de casos confirmados por microbiología o histopatología fue del 44,4 % (24/54), mayor que la prevalencia mundial del 8 % reportada por el *Joint United Nations Programme on HIV/AIDS* (UNAIDS) para el 2021 ^17^. También, supera la cifra del 12,1 % del registro nacional de tuberculosis ^11^, así como las reportadas en Japón ^18^ e Israel ^19^. Incluso, supera las frecuencias documentadas en Europa, en donde se observó un aumento progresivo del porcentaje de pacientes coinfectados -entre el 2007 y el 2016- que pasó del 3 al 12 %; ya para el 2023, la prevalencia de coinfección por tuberculosis pulmonar o extrapulmonar era del 13,6 % ^20^.

Según los datos de la Cuenta de Alto Costo de Colombia del 2024, la prevalencia de coinfección con HIV de los casos registrados de tuberculosis fue del 13,28 %. Asimismo, se reportó que la tamización activa para HIV se realiza entre el 65,6 y el 71,3 % de los casos, según el tipo de aseguramiento al sistema de salud ^21^. La alta frecuencia encontrada en este estudio probablemente se deba a la inclusión únicamente de pacientes hospitalizados y a que la institución es un sitio de referencia en Bogotá para la atención de personas con HIV.

La relación entre hombres y mujeres en el total de casos confirmados fue de 3,9:1, similar a la reportada en otras series de casos publicadas en Bogotá ^22^ y Medellín en Colombia ^23^, y Sao Paulo en Brasil ^24^. No obstante, esta proporción fue menor que la informada en una serie de casos de Chile ^25^ y de otros reportes nacionales previos ^26^^,^^27^. En el subanálisis de los casos confirmados por microbiología o histología, la relación fue ligeramente mayor (4,75:1), cuya comparación con los estudios descritos es similar. La tendencia al incremento de casos en mujeres en los últimos años coincide con lo registrado a nivel global: en el 2023, el 53 % de personas con infección por HIV eran mujeres ^28^.

El nivel de escolaridad fue menor o igual a la básica primaria, en el 44 % de la población total estudiada y en el 50 % de los pacientes con confirmación microbiológica e histopatológica de la infección por tuberculosis. Este factor se relacionó con mayor mortalidad en el estudio de cohorte de Chile (25). Por otra parte, la población extranjera representó el 27,78 % de los casos con cualquier tipo de comprobación diagnóstica y el 37,5 % de quienes tuvieron confirmación microbiológica e histopatológica. Esto también se ha visto relacionado con resultados desfavorables de la enfermedad en Israel y Europa (19,20), debido a las dificultades de acceso a los servicios de salud. Estos datos enfatizan la condición de vulnerabilidad de una parte importante de la población de Bogotá, situación que incrementa los factores de riesgo compartidos para ambas enfermedades e invita a desarrollar estrategias locales colaborativas más robustas, como las instauradas en la región europea ^29^.

Los pacientes tenían un alto grado de inmunosupresión al momento del diagnóstico de la coinfección por *M. tuberculosis* y HIV con el 72,2 % (39/54) en estado 3 según los criterios de los CDC ^16^. De estos, 16 pacientes (29,6 %) tuvieron un número menor o igual a 50 células/μl, datos similares a lo reportado por Cortés *et al*. en Bogotá ^22^, pero mayores a los de Medellín ^23^^,^^27^.

En el grupo de confirmación microbiológica e histopatológica, 17 pacientes (77,3 %) estaban en estado 3 de inmunosupresión. Este factor se asoció con la mortalidad intrahospitalaria en este estudio, hallazgo que se relaciona con lo informado previamente en la literatura científica ^24^^,^^25^.

Entre los factores que probablemente influyeron en el grado de inmunosupresión, se incluyen la proporción de diagnósticos de novo de HIV y que tan solo uno de cada tres pacientes con HIV estaba cumpliendo cabalmente con la terapia antirretroviral. Estas circunstancias están muy por debajo de la meta establecida por ONUSIDA con su estrategia “90-90-90”, la cual busca diagnosticar al 90 % de personas con HIV, que el 90 % tenga acceso a la terapia antirretroviral y que el 90 % tenga supresión de la carga viral ^17^. Las condiciones sociales y educativas desfavorables y la alta prevalencia de pacientes extranjeros (la mayoría sin afiliación al sistema de salud), podrían explicar el pobre cumplimiento de la terapia antirretroviral y el deficiente control inmunovirológico del HIV, factores que deben priorizarse para el diseño y la implementación de medidas de salud pública a nivel nacional y regional.

El 35,2 % (19/54) de los pacientes tuvo un diagnóstico *de novo* de infección por HIV a partir de la sospecha de tuberculosis confirmada por cualquier medio; y, en el caso del grupo confirmado por microbiología o histopatología, fue el 37,5 %. Estos porcentajes resaltan la importancia de la búsqueda activa de coinfección por HIV en pacientes con tuberculosis. Además, el diagnóstico oportuno y el inicio temprano (2 a 4 semanas) de la terapia antirretroviral reducen la mortalidad hasta en el 22 % en este grupo de pacientes ^30^, estando alerta ante la posible aparición del síndrome de reconstitución inflamatoria inmunitaria.

En comparación con quienes tenían un diagnóstico previo de infección por HIV, los pacientes con un diagnóstico *de novo*, tuvieron una mayor carga viral -estadísticamente significativa- así como una mediana de número de linfocitos T CD4^+^ menor y una mayor frecuencia de otras infecciones oportunistas al momento del diagnóstico, aunque sin significancia estadística para estas dos últimas diferencias.

Al considerar todos los pacientes con tuberculosis confirmada de cualquier tipo, la proporción de la forma extrapulmonar fue similar a la informada en estudios locales previamente reportados ^22^^,^^26^, pero menor que la encontrada en otros estudios nacionales ^23^^,^^27^, en los cuales sí hubo similitud en la proporción de casos confirmados mediante microbiología o histopatología. Cabe notar que cerca del 30 % de los pacientes presentó tuberculosis meníngea, valor que se redujo a un 16,7 % en los casos comprobados por laboratorio o patología; aún así, estas proporciones fueron más altas que las documentadas en otros estudios nacionales en los que la presentación ganglionar fue la más frecuente ^22^^,^^23^. Esta última, la ganglionar, también fue la forma extrapulmonar más frecuente en el estudio de caracterización de Meta, Colombia ^31^.

Las tuberculosis extrapulmonares, incluyendo la meníngea, pueden ser todo un desafío diagnóstico. Por ejemplo, la coloración de Ziehl-Neelsen de líquido cefalorraquídeo tiene una sensibilidad del 10 al 15 %, mientras que, el cultivo y las pruebas moleculares -como GeneXpert MTB/RIF o Xpert/RIF Ultra- alcanzan una sensibilidad del 50 al 80 % ^32^. Debido a estas limitaciones diagnósticas, estos estudios no permiten descartar el compromiso meníngeo, por lo cual, en algunos casos, se acude al diagnóstico clínico e imagenológico. En casos de tuberculosis meníngea en pacientes coinfectados con HIV, la mortalidad intrahospitalaria llega a ser de hasta del 60 % ^33^. Por esta razón es necesario tener una gran sospecha clínica de la coinfección e iniciar de forma temprana el tratamiento empírico antituberculoso para evitar desenlaces adversos.

En el presente estudio, 9 de los 16 pacientes con tuberculosis meníngea (56,3 %) -confirmada mediante cualquier método- fallecieron durante su hospitalización, hallazgo que se relaciona con lo encontrado en la literatura científica ^33^^,^^34^, siendo menor (1 de 4) la mortalidad en el subgrupo de pacientes con comprobación microbiológica o histopatológica. En el análisis bivariado con el total de los casos de tuberculosis confirmada, la forma meníngea se identificó como un factor asociado con la mortalidad. Cabe resaltar que la alta prevalencia de tuberculosis meníngea también pudo influir en la baja proporción de confirmaciones microbiológicas observadas en los pacientes.

En total, la mortalidad intrahospitalaria fue del 33,3 %, mayor que la registrada en otros estudios nacionales (9,1 al 20 %) ^22^^,^^23^^,^^27^; mientras que, en los confirmados por microbiología o histopatología fue del 12,5 %. Esto puede estar relacionado con la situación de vulnerabilidad de la población incluida. Los factores asociados con la mortalidad intrahospitalaria fueron tener un diagnóstico reciente de HIV y un bajo número de linfocitos CD4^+^, y presentar tuberculosis meníngea.

Las principales limitaciones de este estudio están relacionadas con su naturaleza retrospectiva, así como con la ausencia de un grupo control para establecer asociaciones estadísticas entre las variables y los resultados de interés. Además, el escaso número de casos confirmados mediante estudio microbiológico o histopatológico no permitió hacer algunos análisis inicialmente propuestos en el estudio. El consecuente alto número de casos tratados con base en criterios clínicos y radiológicos podría estar relacionado con la implementación del estudio de identificación de *M. tuberculosis* mediante prueba molecular para todos los casos, conforme a la resolución 227 de febrero de 2020 de Colombia ^13^. En el periodo previo a dicha resolución, la solicitud de pruebas moleculares dependía del criterio del médico tratante, lo que limitó su realización en la población incluida. Es importante considerar que la sensibilidad de las pruebas moleculares para el diagnóstico de tuberculosis es variable y su disponibilidad es limitada en algunos lugares del país y a nivel mundial, lo que dificulta aún más la confirmación microbiológica.

Para disminuir el sesgo de información, el investigador principal revisó las historias clínicas, identificó las inconsistencias en los registros y resolvió las dudas relacionadas con los criterios de inclusión en conjunto con el médico infectólogo experto. Se necesitan más estudios multicéntricos que actualicen la información sobre las características de la población con tuberculosis y coinfección por HIV, y que incluyan una muestra representativa de pacientes con confirmación microbiológica o histopatológica. El mayor acceso al diagnóstico y el tratamiento tempranos, podría influir en la presentación clínica y los desenlaces de la coinfección. El contar con estos datos permitiría actualizar las medidas de atención en salud pública dirigidas a la población afectada, incluso, haciendo énfasis en la importancia de la confirmación microbiológica o histopatológica del diagnóstico.

En conclusión, la frecuencia de coinfección por *M. tuberculosis* y HIV en nuestro medio es alta y se relaciona con un grado avanzado de inmunosupresión y con varios factores de vulnerabilidad social. Considerando la proporción de pacientes con diagnóstico *de novo* de infección por HIV, en el contexto de una tuberculosis, la búsqueda activa de coinfección puede mejorar la oportunidad en el diagnóstico y el inicio de la terapia antirretroviral. La tuberculosis meníngea fue la forma extrapulmonar más frecuente y se asoció con riesgo de mortalidad, al igual que el escaso número de linfocitos T CD4^+^ y el diagnóstico de novo de infección por HIV.
